# Multiple Sound Sources Localization with Frame-by-Frame Component Removal of Statistically Dominant Source

**DOI:** 10.3390/s18113613

**Published:** 2018-10-24

**Authors:** Maoshen Jia, Yuxuan Wu, Changchun Bao, Jing Wang

**Affiliations:** 1 Beijing Key Laboratory of Computational Intelligence and Intelligent System, Faculty of Information Technology, Beijing University of Technology, Beijing 100124, China; S201761051@emails.bjut.edu.cn (Y.W.); baochch@bjut.edu.cn (C.B.); 2 School of Information and Electronic, Beijing Institute of Technology, Beijing 100081, China

**Keywords:** multiple sound sources localization, direction of arrival estimation, sparsity, soundfield microphone

## Abstract

Multiple sound sources localization is a hot topic in audio signal processing and is widely utilized in many application areas. This paper proposed a multiple sound sources localization method based on a statistically dominant source component removal (SDSCR) algorithm by soundfield microphone. The existence of the statistically weak source (SWS) among soundfield microphone signals is validated by statistical analysis. The SDSCR algorithm with joint an intra-frame and inter-frame statistically dominant source (SDS) discriminations is designed to remove the component of SDS while reserve the SWS component. The degradation of localization accuracy caused by the existence of the SWS is resolved using the SDSCR algorithm. The objective evaluation of the proposed method is conducted in simulated and real environments. The results show that the proposed method achieves a better performance compared with the conventional SSZ-based method both in sources localization and counting.

## 1. Introduction

Multiple sound sources localization is an important topic in audio signal processing and has received a lot of attention in over recent decades [1]. An accurate estimation for direction of arrival (DOA) of a sound source is a vital issue in many applications, such as teleconferences, human-machine interaction, hearing aids [2,3,4] and so on. More and more DOA estimation methods have been developed for exhibiting the ability to provide robustness in adverse conditions such as background noise, interfering sources, and reverberation effects [5,6,7]. Moreover, the information obtained by sound source localization could be applied in spatial sound reproduction [8,9], acoustic analysis of enclosed spaces [10] and spatial audio coding [11,12].

In the early years, the research of DOA estimation mainly focused on the Time Difference of Arrival (TDOA) measurements. TDOAs are usually estimated through peak-picking on the Generalized Cross Correlation of signals acquired at microphone pairs, or on the whole set of microphones [13] and the DOA of sound sources are obtained via the mapping from TDOAs to directions. A series of methods for improving the TDOA estimation have been proposed in References [14,15]. However, most of these TDOA-based localization methods are used at a cost of employing excessive microphones to improve the reliability of TDOA estimation in poorly acoustic conditions, whereas a limited number of microphones are available in practical scenarios [16]. 

The multiple signal classification (MUSIC) algorithm is one of the well-known subspace-based methods for estimating DOAs of multiple sources in overdetermined conditions (i.e., microphones number more than sources number) [17,18], which depends on the eigen-decomposition for the covariance matrix of observation vectors. Another popular subspace-based method for DOA estimates is estimation of signal parameters by rotational invariance techniques (ESPRIT), which is more robust to array imperfections than MUSIC because it exploits the rotational invariance property in the signal subspace created by two subarrays [19]. Nevertheless, the main drawbacks of subspace-based methods for DOA estimation lie in the computational cost and the number of microphones. Moreover, maximizing the steered response power (SRP) of a beamformer is also used to estimate DOAs of multiple sources. However, SRP-based methods are highly demanding in terms of computational complexity due to performing a time-consuming search process over some space [20].

Recently, Independent component analysis (ICA) has been adopted in DOA estimation by directional sparsity of sound sources [21,22,23]. Moreover, Reference [16] proposes implementing ICA in time-frequency (T-F) domain for multiple sound sources localization under the condition that the number of dominant sources did not exceed the number of microphones in each T-F zone. Similarly, sparse component analysis (SCA) based methods for estimating DOAs of multiple sources [24,25] are proposed under the W-Disjoint Orthogonality (W-DO) assumption that the effect of one source is dominant compared with the other sources in some T-F bins. Following this assumption, the problem of multiple sources localization might be solved by single source DOA estimation for each T-F bin. For example, a recent method based on the W-DO assumption has been proposed which achieved a high localization accuracy by a pair of coincident soundfield microphones (i.e., B-format microphone) [26]. Most of the SCA-based methods are dependent on the W-DO property of multiple sound sources meaning that respective time-frequency representations of sources are located in different T-F bins. However, when the number of simultaneously occurring sources are four or above, more than one source is active in a T-F bin with a high probability. It means that this assumption is less accurate when the number of sound sources increases, which would also affect the localization accuracy of the SCA-based method.

To address this issue, a localization method that applied the relaxed sparsity constraints of multiple sound sources has been proposed in Reference [27]. The method bases on an assumption that several T-F bands always exist among signals recorded by the circular microphone array (CMA-based method), where one source is dominant over others. That is, some single source zones (SSZ) can be found when multiple sound sources occurred simultaneously. Under this assumption, the DOA estimation could be proceeded in these zones to improve the localization accuracy. Some evaluation experiments show that the method is slightly better than other localization methods, both in accuracy and computational complexity. However, a high localization accuracy of this method can be achieved under the case that the number of sources is no more than the number of microphones. It means that as the number of simultaneously occurring sound sources increase, excessive microphones are required to ensure a reliable accuracy. To solve this problem, an improvement of this DOA estimation method has been proposed [28] to get a high accuracy of multiple sound sources localization by soundfield microphone. In this paper, the localization method proposed in Reference [28] is referred to as the SSZ-based method. Generally, the SSZ-based method estimates the DOA locally in each T-F bin contained in the detected SSZ and associates these DOAs to each source by means of a histogram or clustering. Nevertheless, as the number of simultaneously occurring sources increasing (six or above), some sound sources are difficult to detect due to the contribution of these sources component that are obviously weaker than others in the obtained histogram. The comment of these sound source localization methods mentioned above are summarized in Table 1.

In this paper, we make an investigation for the problem SSZ-based method faced in the scene that multiple sound sources simultaneously occur. According to the results shown in the investigation, it is confirmed that there is a phenomenon where a sound source with less associated DOA estimates in the histogram is difficult to detect. Therefore, these sources are defined as statistically weak sources (SWS), while other sources are considered to be statistically dominant sources (SDS). In order to find out the SWS to improve localization performance, we present a multiple sound sources localization method based on statistically dominant source component removal (SDSCR) algorithm in this paper. For the SDSCR algorithm, we design an intra-frame SDS discriminator and inter-frame SDS discriminator to divide the DOA estimates of each T-F bin into SWS component and SDS component. Then, removal processing is designed to retain the SWS components and remove the SDS components. Finally, the estimated azimuth can be obtained by clustering the selected DOA estimates. After the localization performance evaluation in simulated and real environments, the proposed method shows good performance both in DOA estimation and sources counting.

The key contributions of this paper can be summarized as follows. The existence of the SWS in the different number of sources simultaneously occurring scenario is investigated and validated. For dealing with the phenomenon that the SWS cannot be detected by the conventional SSZ-based method, the multiple sound sources localization method based on the SDSCR algorithm is proposed here. Compared with the SSZ-based method, the proposed method achieves robust performance of multiple sound sources localization via the SDSCR algorithm.

The remainder of the paper is organized as follows: Section 2 states problem in the SSZ-based method and investigates the existence of the SWS among soundfield microphones signals. Section 3 introduces the proposed SDSCR algorithm for multiple sound sources localization. Experimental results are presented in Section 4, while conclusions are drawn in Section 5.

## 2. Problem Statement

### 2.1. Problem in the SSZ-Based Method

**Figure 1:**
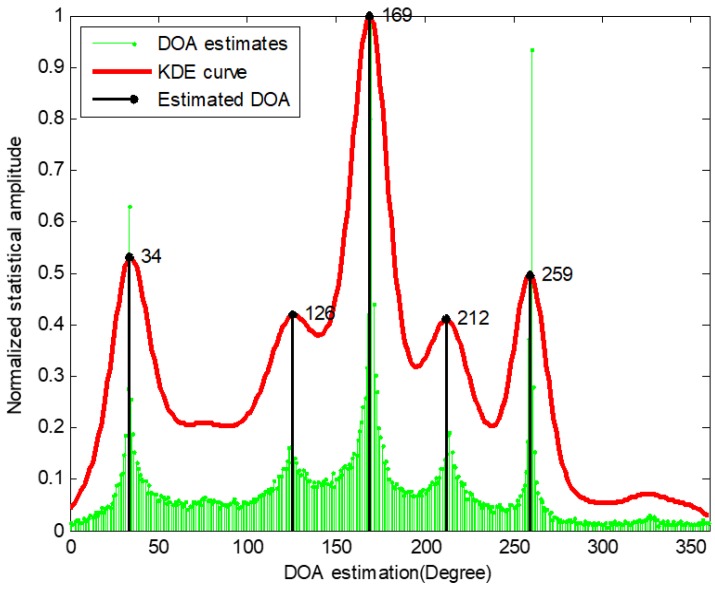
Normalized amplitude DOA estimation histogram of seven sources.

In general, the SSZ-based method which associates DOAs to each source by means of a clustering consists of three steps. The first step is searching SSZ by using the signals correlation between different channels of soundfield microphone. In the second step, a histogram of DOA estimates calculated from all detected SSZs is obtained by statistical analysis. Finally, azimuths of all sound sources are estimated after a series of post-processing. The SSZ-based method generally exhibits a good performance for multiple sound sources localization, but as the sound sources number increases, the SSZ-based method exposes a problem that affect localization accuracy, especially when the number of sound sources is greater than six. For instance, we conducted an experiment to illustrate this issue. In this test, a soundfield microphone is used for recording seven sound sources respectively located in 35°, 75°, 125°, 170°, 210°, 260°, and 325° in an anechoic room. The normalized amplitude DOA estimation histogram obtained by the SSZ-based method is shown in Figure 1. The green stem is the histogram of DOA estimates that employs T-F bins within all detected SSZs and the red curve is the estimated envelope of histogram proceeded by kernel density estimation (KDE). From Figure 1, it can be found that there are only five obvious peaks (around 35°, 125°, 170°, 210° and 260°) in the KDE curve that can be found, while the other two peaks around 75° and 325° cannot be detected by means of peak searching processing. A sound source with more associated SSZs (or DOA estimates) has an obviously higher normalized amplitude at the corresponding peak in a DOA histogram, meaning that the azimuth of this sound source has a higher probability of occurrence in all obtained DOA estimates. On the contrary, some sources with less associated SSZs have obviously lower normalized amplitude and become more difficult to search for in a DOA histogram. For Figure 1, the sound source located in 75° and 325°cannot be detected in the obtained histogram due to only a small amount of SSZs being associated with the two sound sources compared with other five sound sources. Additionally, from an informal experiment, we find that if the number of SSZs are associated with one sound source less than 4% of the total number of SSZs, the peak corresponding to these sources cannot be detected in the DOA estimation histogram. Generally, in this work, a sound source with less associated DOA estimates in the histogram is referred as to a statistically weak source (SWS), while the other sources are referred as statistically dominant sources (SDS). It can be clearly seen that the performance of the SSZ-based method in sources counting is degraded due to the missed detection of SWSs. Additionally, we consider a result of a sound source with missed detection as a large deviation between the estimated DOA and the true DOA. It means that the missed detection of SWSs also degrades the performance of the SSZ-based method in DOA estimation. Therefore, the problem SSZ-based method faced is the localization accuracy decreasing when the SWS exists, meaning that it is difficult to detect the SWSs among soundfield microphone signals using the SSZ-based method. To solve this problem, we consider retaining the SSZs that are associated with the SWS while filtering out the SSZs that are associated with other sound sources. More details about the implementation process of the presented solution are described in Section 3. Then we conduct a statistical analysis for the existence of the SWS among signals recorded by soundfield microphone and statistical analysis results are given in Section 2.2.

### 2.2. Exploring the Swss among Microphone Signals 

**Figure 2:**
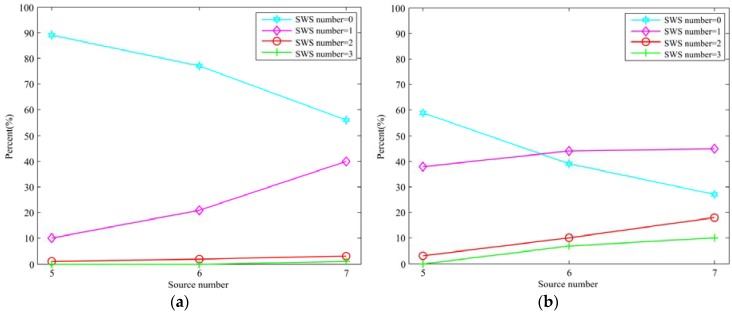
Statistical analysis for the existence of the SWS in different sound source number (**a**) Angle between sources is 50°; (**b**) Angle between sources is 30°.

The problem caused by the SWSs has been pointed out above. We then aim to investigate the existence of the SWS in the different number of sources with simultaneously occurring scenarios. For examining the existence of the SWS**,** some statistical analysis were taken in an anechoic room and Room 1 (250 ms reverberation time room), respectively. The speech signals from the NTT database were chosen as sound sources for analysis. The recording was simulated by Roomsim [29]. The angle between sources were set as γ = {30°, 50°} and γ = 50° in the anechoic room and Room 1, respectively. Meanwhile, the sources number was ranging in {5, 6, 7} and {3, 4, 5} in the anechoic room and Room 1, respectively. More specifically, for each sources number and angle, we selected 100 segments of data and were satisfied that all sound sources simultaneously occurring in the time domain and at least half of the sound sources are detected accurately by the SSZ-based method to make the statistical analysis. Moreover, a sound source was considered as the SWS when the sound source was lost in detection or the localization error was more than 5 degrees. We collect the percentage of the SWS number over the total 100 valid statistics, and the results are shown in Figure 2a,b that represent the statistical results of the angle between sources are 30° and 50° in anechoic room, respectively.

From Figure 2, a visualized conclusion can be drawn that the existence probability of the SWS has a positive correlation and a negative correlation with the number of sound sources and the angle between sources, respectively. More specifically, as sound sources number increases, the number of SWSs increases (i.e., more sources cannot be detected). For example, from Figure 2a, the angle between sources was set as 50°, the probability of no SWS (i.e., the number of SWS is zero, all sources can be detected) is nearly 90% when the sound sources number was set as five and the probability of no SWS drops to 70% and 59%, when the sound sources number was six and seven, respectively. On the contrary, the probability of one SWS and two SWSs rises with the increase of sound sources number, which means that as the number of sound sources increases, the existence probability of the SWS rises (i.e., the number of SWSs increase). 

From another perspective, as the angle between sources decreases, the number of SWS increases. For instance, from Figure 2a,b, the number of sound sources was set as seven, when the angle between sources was set as 50°, the probability of no SWS is 56% and when the angle between sources was set as 30°, the probability of no SWS drops to 27%. The probability of one SWS and two SWSs increases from 40% to 45% and from 3% to 18%, respectively, when the angle between sources drops from 50° to 30°.

In general, from the perspective of sound sources number, when the sound sources number is five, there have no SWS with a high probability. Nevertheless, the probability of occurrence of the SWS is obviously increasing when the sound sources number is six. The existence probability of one SWS is higher than that of no SWS when the sound sources number is seven, which means that the SWS almost always exist. 

**Figure 3:**
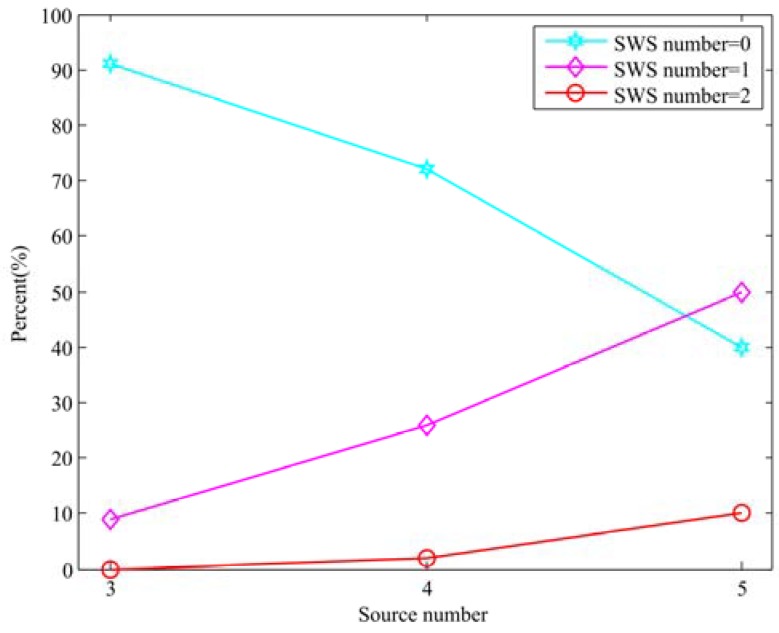
Statistical analysis for the existence of the SWS in Room 1.

In order to investigate the existence of the SWS in different scene, we collect the percentage of SWSs number with different sources number in the Room 1 (250 ms reverberation time), the angle between sources is 50° in this experiment. The results are shown in Figure 3. From Figure 3, it is clear that as the number of sound sources increase from three to five, the existence probability of the SWS increases. We can find similar conclusions as the experiment results in the anechoic condition.

From the above experimental results, it can be summarized that the number of SWSs varies with the number of sound sources and the angle between sources. The SWS is ubiquitous, however, the existing SWS affects the accuracy of multiple sound sources localization method which exploit all SSZs for DOA estimation. For addressing the problem introduced by the SWS, we proposed the SDSCR algorithm with joint the intra-frame and inter-frame SDS discrimination. More details about the SDSCR algorithm will be described in the next section.

## 3. Proposed Method

**Figure 4:**
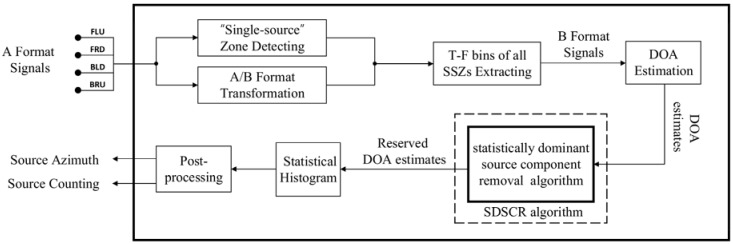
The block diagram for the proposed multiple sound sources localization framework.

From the conclusion of Section 2, it can be obtained that the SWS is ubiquitous in the scenario where multiple sound sources occur simultaneously. This phenomenon is particularly evident for the signals recorded by soundfield microphone. In this section, we present the SDSCR algorithm for multiple sound sources localization using a soundfield microphone, for solving the problem caused by the SWS existing in the detected SSZs. The following process is performed on a frame-by-frame basis. The system block diagram of the proposed scheme is shown in Figure 4. Input A-format signals are transformed into the T-F domain using short-time Fourier transform (STFT) for single-source zone detecting, and B-format signals are obtained from A-format signals by (1). If single-source zones exist in the current frame, the B-format T-F bins of all SSZs need to be extracted for DOA estimation. Thereafter, we present the SDSCR algorithm with joint intra-frame and inter-frame SDS discrimination to remove the partial DOA estimates that are associated with the SDS. The histogram of selected DOA estimates is obtained by statistical analysis. Finally, the number of sources and their DOA estimates are as output after the post-processing process. More details of these processes will be described below.

### 3.1. Review of DOA Estimation Based on the SSZ Detecting

The investigation of Reference [28] has indicated that for recordings of sound scenes where multiple sound sources occur simultaneously, there must be several T-F zones where only one source is active. These T-F zones are referred to as single-source zones. Therefore, a highly accurate DOA estimation and sources counting approach for multiple sound sources localization by conducting the DOA estimation in these SSZs has been proposed in Reference [28]. A soundfield microphone consists of four co-located microphones placed at the four non-adjacent corners of a cube [30] which are referred to as Front Left Up (FLU), Front Right Down (FRD), Back Left Down (BLD), and Back Right Up (BRU) microphones, respectively. The raw signals recorded by soundfield microphone are called A-format signal i.e.,$\;\left\{S_{FLU}\left(n,k\right),S_{FRD}\left(n,k\right),S_{BLD}\left(n,k\right),S_{BRU}\left(n,k\right)\right\}$, where *n* and *k* represent the frame number and the frequency index, respectively. The A-format signals are transformed to B-format which consists of one omnidirectional (W) and three figure-of-eight directional (X, Y, Z) channels, i.e., $\left\{S_{W}\left(n,k\right),S_{X}\left(n,k\right),S_{Y}\left(n,k\right),S_{Z}\left(n,k\right)\right\}$ [31,32] as follows:

(1) $$\left\{ \begin{array}{l} S_{W}\left(n,k\right)=S_{FLU}\left(n,k\right)+S_{FRD}\left(n,k\right)+S_{BLD}\left(n,k\right)+S_{BRU}\left(n,k\right)\\ S_{X}\left(n,k\right)=S_{FLU}\left(n,k\right)+S_{FRD}\left(n,k\right)-S_{BLD}\left(n,k\right)-S_{BRU}\left(n,k\right)\\ S_{Y}\left(n,k\right)=S_{FLU}\left(n,k\right)-S_{FRD}\left(n,k\right)+S_{BLD}\left(n,k\right)-S_{BRU}\left(n,k\right)\\ S_{Z}\left(n,k\right)=S_{FLU}\left(n,k\right)-S_{FRD}\left(n,k\right)-S_{BLD}\left(n,k\right)+S_{BRU}\left(n,k\right) \end{array}\right.$$

A speech source is known to be sparse in the short-term T-F domain [26]. Therefore, the detection of SSZs existing among the soundfield microphone signals is implemented in the T-F domain. Due to the directional characteristics of the soundfield microphone, when there is only one active source within some T-F zones, the raw signals recorded in each channel will have a strong correlation. In contrast, for zones where multiple active sources occur simultaneously, the correlation between the recorded signals will be weaker. Therefore, a generalized multichannel cross-correlation coefficient [33,34] (G-MCCC) is defined to measures the correlation of recorded A-format signal among the four channels. 

For obtaining the G-MCCC in a T-F zone, in detail, the full frequency band consists of *K* STFT bins for a frame is divided into *C* zones $L_{c}$. $L_{c}$ is a set of adjacent T-F points corresponding to a sub-band of STFT coefficients (*c* ∈ [1, *C*] and for simplicity $L_{c}$ is represented by L in this section). In addition, we take $\left\{S_{1}\left(n,k\right),S_{2}\left(n,k\right),S_{3}\left(n,k\right),S_{4}\left(n,k\right)\right\}$ to represent the A-format recorded signals $\left\{S_{FLU}\left(n,k\right),S_{FRD}\left(n,k\right),S_{BLD}\left(n,k\right),S_{BRU}\left(n,k\right)\right\}$ for simplicity.

More specifically, for any pair of soundfield microphone recorded signals $S_{i}\left(n,k\right)$ and $S_{j}\left(n,k\right)$, a multichannel cross-correlation coefficient is defined as:

(2) $$\;R_{ij}\left(L\right)=\underset{\left(n,k\right)\in L}{\sum }\left|S_{i}\left(n,k\right)\cdot S_{j}\left(n,k\right)\right|\;$$

where i≠j,$\;S_{i}\left(n,k\right),S_{i}\left(n,k\right)\in \left\{S_{1}\left(n,k\right),S_{2}\left(n,k\right)S_{3}\left(n,k\right),S_{4}\left(n,k\right)\right\}$. Then the G-MCCC can be obtained by

(3) $$\;r_{ij}\left(L\right)=\frac{R_{ij}\left(L\right)}{\sqrt{R_{ii}\left(L\right)\cdot R_{jj}\left(L\right)}}\;$$

A necessary and sufficient condition for a zone L to be a single-source zone is

(4) $$\;r_{ij}\left(L\right)=1\;$$

where i∈{1, 2, 3, 4}, j=i+1 mod 4 and mod is a function for remainder operation. We search for single-source zones that satisfy the following inequality:

(5) $$\;r_{ij}\left(L\right)>1-\varepsilon \;$$

where ε is a sufficiently small threshold for a user to define and the value of ε is generally less than 0.1. The DOA estimates $\hat{\mu }\left(n,k\right)$ of each T-F bin in a single-source zone $L_{s}$ can be calculated by processing the B-format signals (obtained from the four channel signals):

(6) $$\;\hat{\mu }\left(n,k\right)={tan}^{-1}\left(\frac{Re\left\{S_{W}{}^{*}\left(n,k\right)\cdot S_{Y}\left(n,k\right)\right\}}{Re\left\{S_{W}{}^{*}\left(n,k\right)\cdot S_{X}\left(n,k\right)\right\}}\right)\;$$

where *Re*{ · } denotes taking the real part of the argument and * denotes conjugation. Both the SWS and SDS components are present in DOA estimates. The purpose of the SDSCR algorithm is to remove the component of some SDSs so that the SWS components can be found using histogram statistics. The SDS discrimination within the SDSCR algorithm consists of two steps, one is intra-frame SDS discrimination, the other is inter-frame SDS discrimination. More details about the SDSCR algorithm are presented in the following Sections.

### 3.2. SDSCR Algorithm 

Based on the investigation in Section 2, the conclusion can be drawn that the SWS, which is ubiquitous in the multiple sound sources occurring simultaneously scenario, is difficult to detect by the conventional SSZ-based DOA estimation method. Therefore, for dealing with the degradation of localization accuracy caused by the existence of the SWS, we propose an SDSCR algorithm to remove the component of SDSs while preserves the SWSs component in each frame.

**Figure 5:**
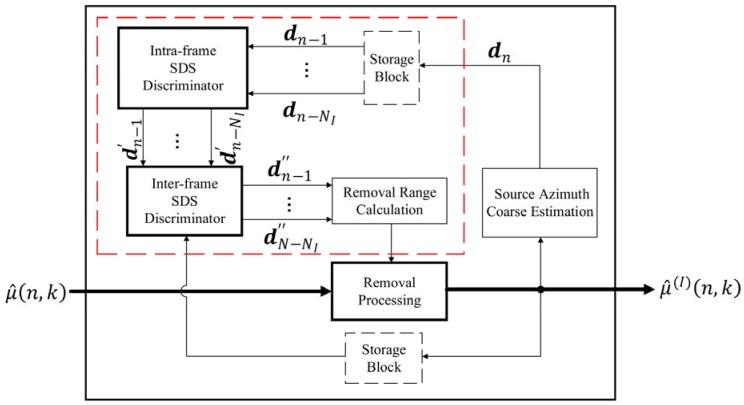
The block diagram of the components removal with joint intra-frame and inter-frame SDS discrimination.

The SDSCR algorithm with joint the intra-frame and inter-frame SDS discrimination is proposed in this section as described by block diagram of Figure 5. For the current frame, the DOA estimates of each T-F bin in all detected SSZ (i.e., $\hat{\mu }\left(n,k\right)$) are chosen as input information for SDSCR algorithm. Then the selected DOA estimates (defined as ${\hat{\mu }}^{\left(I\right)}\left(n,k\right)$) of current frame are obtained by filtering out some $\hat{\mu }\left(n,k\right)$ locating in the removal range in removal processing. Finally, the selected DOA estimates are used for obtaining the histogram statistics to estimate the azimuth of each source. In addition, a source azimuth coarse estimation of current frame (i.e., $d_{n}$) is estimated for guiding later frames to calculate the removal range by using ${\hat{\mu }}^{\left(I\right)}\left(n,k\right)$.

The calculation process of removal range shown in the red dashed box of Figure 5 is realized with the historical source azimuth coarse estimation information (i.e., the source azimuth coarse estimation of look-ahead frames). More specifically, the intra-frame SDS discriminator is used for obtaining the azimuth where the intra-frame SDS is considered as locating. Then, the intra-frame SDS is further discrimination in the inter-frame SDS discriminator to preserve the reasonable azimuth of SDS while eliminating the azimuth that is not the SDS locating. Finally, the removal range of current frame is calculated by the discriminated SDS.

For the removal range calculation, the two core operations are intra-frame SDS discrimination and intra-frame SDS discrimination, respectively. In general, the purpose of the intra-frame SDS discriminator is searching the intra-frame SDS in each frame. More specifically, azimuth information of a sound source can be calculated for each SSZ, while there are multiple SSZs in a frame with a high probability. In a frame, different sound sources are detected with different times which means that the probability of occurrence of each detected sound source is different in the frame. Therefore, the core idea of the intra-frame SDS discriminator is divided sound sources detected in the frame into the intra-frame SDS and SWS according to the probability of occurrence of each source. According to this phenomenon, the intra-frame SDS discrimination is designed (shown in algorithm1). The input of the intra-frame SDS discriminator are the historical source azimuth coarse estimation information $d_{n-1},\ldots ,\;d_{n-N_{I}}$, where $d_{n-i}=\left(d_{1}^{n-i},d_{2}^{n-i},\ldots ,d_{N_{n-i}}^{n-i}\right),\;i=1,\;2,\ldots ,N_{I},$ is a vector including the coarse estimation of azimuth for each valid SSZ (i.e., at least half of DOA estimates are not removed in the SSZ) in the (*n − i*)th frame. $N_{I}$ is the number of look-back frames used to calculate the removal range, $d_{N_{n-i}}^{n-i}$ and $N_{n-i}$ are azimuth coarse estimate and the number of valid SSZs in the (*n − i*)th frame (i.e., the number of azimuth coarse estimates), respectively.

For obtaining a reasonable intra-frame SDS, it should be noted that there are two thresholds which are referred as to the minimum difference threshold $\varepsilon_{a}$ and the minimum distance threshold $\varepsilon_{b}$ need to be set. In details, the $\varepsilon_{a}$ and $\varepsilon_{b}$ are used to count the number of sound sources and intra-frame SDS, respectively. The output of the intra-frame SDS discriminator are ${d^{\prime }}_{n-i}=\left({d^{\prime }}_{1}^{n-i},{d^{\prime }}_{2}^{n-i},\ldots ,{d^{\prime }}_{N_{n-i}^{\prime }}^{n-i}\right)$, where ${d^{\prime }}_{n-i}$ is a vector consists of intra-frame SDS azimuths in the (*n-i*)th frame and $N_{n-i}^{\prime }$ is the number of elements in ${d^{\prime }}_{n-i}$. Moreover, ${d^{\prime }}_{n-i}$ are the input of the inter-frame SDS discriminator. 

The discriminated SDS by Algorithm 1 described above is the intra-frame SDS which is obtained without the historical information (DOA estimates in historical frames). More specifically, among all reserved DOA estimates, there may be only a few DOA estimates that are associated with an intra-frame SDS in the current frame (i.e., only a few DOA estimates are in the same direction as the SDS). These intra-frame SDSs are referred as pseudo-SDSs or local-SDSs and the azimuth of these intra-frame SDSs need to be excluded to form the removal range. Therefore, the inter-frame SDS discriminator is designed to filter out the pseudo-SDS, for avoiding that the azimuth of the pseudo-SDS is within the removal range. The basic idea of the inter-frame SDS discriminator is searching the pseudo-SDS by exploiting the number of DOA estimates that are associated with the intra-frame SDS. The inter-frame SDS discrimination proceeds are shown as Algorithm 2. Similar to the Algorithm 1, two thresholds the removal range threshold Δμ and the minimum quantity threshold $\varepsilon_{c}$ need to be set in the inter-frame SDS discriminator. Specifically, if the DOA of the *i*th source is $\mu_{i}$, a range around $\mu_{i}$ (i.e., [$\mu_{i}-\Delta \mu \;,\;\mu_{i}+\Delta \mu$]) needs to be determined such that the DOA estimates within this range are considered to be derived from source *i* and $\varepsilon_{c}$ is used to filter out the azimuth of pseudo-SDS.

The output of the inter-frame SDS discriminator are ${d^{\prime \prime }}_{n-1},\ldots ,\;{d^{\prime \prime }}_{n-N_{I}}$. ${d^{\prime \prime }}_{n-i}=\left({d^{\prime \prime }}_{1}^{n-i},{d^{\prime \prime }}_{2}^{n-i},\ldots ,{d^{\prime \prime }}_{N_{n-i}^{\prime \prime }}^{n-i}\right)$ are used to calculate the removal range $D=\cup_{i=1}^{N_{I}}D_{n-i}$, where $D_{n-i}=\cup_{j=1}^{N_{n-i}^{\prime \prime }}D_{n-i}^{j}$ and $D_{n-i}^{j}=\left[{d^{\prime \prime }}_{j}^{n-i}-\Delta \mu ,{d^{\prime \prime }}_{j}^{n-i}+\Delta \mu \;\right]$. If $\hat{\mu }\left(n,k\right)$ does not locating in the removal range (i.e., $\hat{\mu }\left(n,k\right)\;\notin D$), the selected DOA estimate ${\hat{\mu }}^{\left(I\right)}\left(n,k\right)$ is obtained as follow:

(7) $$\;{\hat{\mu }}^{\left(I\right)}\left(n,k\right)=\hat{\mu }\left(n,k\right)\;$$

On the contrary, if $\hat{\mu }\left(n,k\right)$ locates in the removal range, ${\hat{\mu }}^{\left(I\right)}\left(n,k\right)$ is defined as an illegal element.

In addition, the source azimuth coarse estimation vector in the *n*th frame ($d_{n}$) is obtained by ${\hat{\mu }}^{\left(I\right)}\left(n,k\right)$. More specifically, in the *n*th frame, for the searched single-source zone $L_{s}$, ${\hat{\mu }}^{\left(I\right)}\left(L_{s}\right)$ is a vector consist of ${\hat{\mu }}^{\left(I\right)}\left(n,k\right)$ where $\left(n,k\right)\in L_{s}$. The validity of each ${\hat{\mu }}^{\left(I\right)}\left(L_{s}\right)$ is calculated and the invalid ${\hat{\mu }}^{\left(I\right)}\left(L_{s}\right)$ (i.e., more than half of the ${\hat{\mu }}^{\left(I\right)}\left(n,k\right)$ in $\;{\hat{\mu }}^{\left(I\right)}\left(L_{s}\right)$ are illegal elements) are filtered out. The azimuth coarse estimation is obtained by calculating the median of reserved ${\hat{\mu }}^{\left(I\right)}\left(L_{s}\right)$. For each valid single-source zone $L_{s}$, we calculate the azimuth coarse estimation and formed a vector $d_{n}$. 

**Algorithm 1**: Intra-frame SDS Discrimination**Input**: $N_{I}$        ►The number of look-back frames used to calculate the removal range **Input**: $d_{n-1},\ldots ,\;d_{n-N_{I}}$, where $d_{n-i}=\left(d_{1}^{n-i},d_{2}^{n-i},\ldots ,d_{N_{n-i}}^{n-i}\right)$, $i=1,\;2,\ldots ,N_{I}$ **Output**: ${d^{\prime }}_{n-1},\ldots ,\;{d^{\prime }}_{n-N_{I}}$, where ${d^{\prime }}_{n-i}=\left({d^{\prime }}_{1}^{n-i},{d^{\prime }}_{2}^{n-i},\ldots ,{d^{\prime }}_{N_{n-i}^{\prime }}^{n-i}\right)$, $i=1,\;2,\ldots ,N_{I}$ 1. **for***i* = 1 to $N_{I}$
**do** 2. Sort azimuth coarse estimates (the elements in $d_{n-i}$) in ascending order  $d_{n-i}^{sort}=\left(d_{a_{1}}^{n-i},d_{a_{2}}^{n-i},\ldots ,d_{a_{N_{n-i}}}^{n-i}\right)$, where $a_{1}$,$\;a_{2}$,…,$\;a_{N_{n-i}}\in$ [1,$\;N_{n-i}$] are new index after sorting                                ►$d_{a_{1}}^{n-i}$ is the minimum element in $d_{n-i}$                                ►$d_{a_{N_{n-i}}}^{n-i}$ is the maximum element in $d_{n-i}$ 3. **for**
*j* = 1 to $N_{n-i}-1$
**do**   ►look for the number of sound sources in $d_{n-i}^{sort}$ 4.  **if**
$\left|d_{a_{j+1}}^{n-i}-d_{a_{j}}^{n-i}\right|>\varepsilon_{a}$, i.e., two estimated azimuths come from different source                                ►$\varepsilon_{a}$ is the minimum difference threshold 5.   **then**
$m_{j}=j$, **else** Set $m_{j}$ to be an illegal element:$\;m_{j}=0$ 6.  **end if** 7. **end for** 8. $m=\left(m_{1},m_{2},\ldots ,m_{N_{n-i}-1}\right)$ 9. $M={\left\|m\right\|}_{0}$ 10. **if**
M≠0, i.e., all azimuths come from the different sound sources in $d_{n-i}^{sort}$ 11.  Delete the illegal elements in the vector m and form a new index vector $m^{\prime }=\left(m_{1}^{\prime },m_{2}^{\prime },\ldots ,m_{M}^{\prime }\right)$, 12.  **for**
*k* = 1 to M
**do**   ►look for the intra-frame SDS 13.   **if**
$\left|m_{k}^{\prime }-m_{k-1}^{\prime }\right|>\varepsilon_{b}$,   i.e., $d_{a_{m_{k-1}^{\prime }+1}}^{n-i},\ldots ,\;d_{a_{m_{k}^{\prime }}}^{n-i}$ come from the same SDS                                ►$\varepsilon_{b}$ is the minimum distance threshold 14.   **then**
$d_{k}^{\prime }=\frac{\sum_{p=m_{k-1}^{\prime }+1}^{m_{k}^{\prime }}d_{a_{p}}^{n-i}}{m_{k}^{\prime }-m_{k-1}^{\prime }-1}$, 15.   **else** set $d_{k}^{\prime }$ to be an illegal element: $d_{k}^{\prime }=-1$ 16.   **end if** 17.  **end for** 18.  $d^{temp}=\left(d_{1}^{\prime },d_{2}^{\prime },\ldots ,d_{M}^{\prime }\right)$   Delete the illegal elements in $d^{temp}$ and form the vector ${d^{\prime }}_{n-i}=\left({d^{\prime }}_{1}^{n-i},{d^{\prime }}_{2}^{n-i},\ldots ,{d^{\prime }}_{N_{n-i}^{\prime }}^{n-i}\right)$   where $N_{n-i}^{\prime }$ is the number of elements in ${d^{\prime }}_{n-i}$ (i.e., the number of estimated intra-frame SDS) 19. **else**, i.e., M=0, all azimuths come from the same sound source in $d_{n-i}^{sort}$ 20.  Vector ${d^{\prime }}_{n-i}$ consists of the largest and smallest elements in $d_{n-i}^{sort}$ 21. **end if** 22. **end for** 23. **Output:**
${d^{\prime }}_{n-1},\ldots ,\;{d^{\prime }}_{n-N_{I}}$

**Algorithm 2**: Inter-frame SDS Discrimination**Input**: ${d^{\prime }}_{n-1},\ldots ,\;{d^{\prime }}_{n-N_{I}}$, where ${d^{\prime }}_{n-i}=\left({d^{\prime }}_{1}^{n-i},{d^{\prime }}_{2}^{n-i},\ldots ,{d^{\prime }}_{N_{n-i}^{\prime }}^{n-i}\right)$, $i=1,2,\ldots ,N_{I}$ **Output**: ${d^{\prime \prime }}_{n-1},\ldots ,\;{d^{\prime \prime }}_{n-N_{I}}$, where ${d^{\prime \prime }}_{n-i}=\left({d^{\prime \prime }}_{1}^{n-i},{d^{\prime \prime }}_{2}^{n-i},\ldots ,{d^{\prime \prime }}_{N_{n-i}^{\prime \prime }}^{n-i}\right)$, $i=1,2,\ldots ,N_{I}$                     ►${d^{\prime \prime }}_{n-i}$ is a set of estimated SDS azimuths in the (*n* − *i*)th frame                                ►$N_{n-i}^{\prime \prime }$ is the number of SDSs in ${d^{\prime \prime }}_{n-i}$ Count the number of DOA estimates of all history frames and record as $N_{DEPs}$ 1. **for**
*i* = 1 to $N_{I}$
**do** 2. **for**
*j* = 1 to $N_{n-i}^{\prime }$
**do**  Count the number of DOA estimates of all history frames locating in [${d^{\prime }}_{j}^{n-i}$ − Δμ, ${d^{\prime }}_{j}^{n-i}$ + Δμ]                                        and record as $N_{DEPs}^{\prime }$                                 ►Δμ is the removal range threshold 3.  **if**
$\frac{N_{DEPs}^{\prime }}{N_{DEPs}}>\varepsilon_{c}$   It means that there are enough DOA estimates belonging to azimuth ${d^{\prime }}_{j}^{n-i}$                    corresponding sound source and the sound source is judged as SDS 4.  **then,**
$d_{j}^{G}={d^{\prime }}_{j}^{n-i}$ 5.  **else** 6.   Set $d_{j}^{G}$ as an illegal element: $d_{j}^{G}=-1$ 7.  **end if** 8. **end for** 9. $d^{G}=\left(d_{1}^{G},d_{2}^{G},\ldots ,d_{N_{n-i}^{\prime }}^{G}\right)$  Delete the illegal elements in the set $d^{G}$ and form vector ${d^{\prime \prime }}_{n-i}=\left({d^{\prime \prime }}_{1}^{n-i},{d^{\prime \prime }}_{2}^{n-i},\ldots ,{d^{\prime \prime }}_{N_{n-i}^{\prime \prime }}^{n-i}\right)$, 10. **end for** 11. **Output:**
$\;{d^{\prime \prime }}_{n-1},\ldots ,\;{d^{\prime \prime }}_{n-N_{I}},\;\;i=1,\;2,\ldots ,N_{I}$

The histogram can be obtained by clustering all legal ${\hat{\mu }}^{\left(I\right)}\left(n,k\right)$. As a result, the SWSs are more easily searched due to the components of a mass of DOA estimates that are associated with the SDSs are removed by the SDSCR algorithm in the DOA estimation histogram.

**Figure 6:**
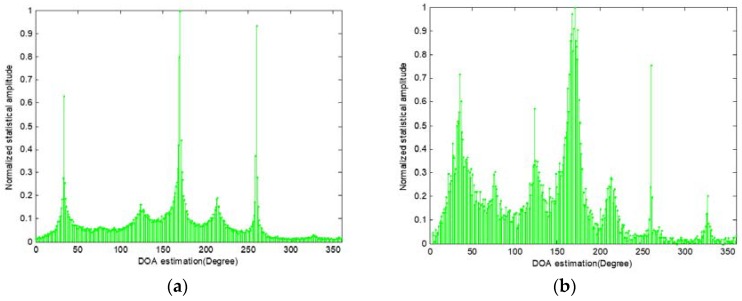
Normalized amplitude histogram of seven sources (**a**) DOA estimation exploit SSZ-based method; (**b**) DOA estimation exploit SDSCR algorithm.

An example of the normalized histogram of recorded signals consist of seven sources locating at 35°, 75°, 125°, 170°, 210°, 260° and 325° is shown in Figure 6, where the histogram of all $\hat{\mu }\left(n,k\right)$ and ${\hat{\mu }}^{\left(I\right)}\left(n,k\right)$ are shown in Figure 6a,b, respectively. The soundfield microphone signals were recorded in an anechoic room, the width of single-source zone was 64, $N_{I}$ and Δμ were 3 and 3° respectively. The length of data was 1s. According to the results shown in Figure 6a,b, it can be clearly seen that the proposed method finds two sound sources that are missed by the SSZ-based methods, i.e., the SWSs can be detected by the proposed method. More specifically, from Figure 6a, there are only three obvious peaks which are around 35°, 170° and 210°, while the normalized amplitude of peaks around 125° and 260° are obviously lower than other three peaks. It should be noted that the peaks which should appear around 75° and 325° are lost in the DOA histogram. This phenomenon means that the existing SWSs affect the accuracy of conventional SSZ-based method for multiple sound sources localization seriously. Contrary to the results shown in Figure 6a where two sources are lost, in Figure 6b, two peaks have already observed around 75° and 325° by exploiting the SDSCR algorithm. Meanwhile, the normalized amplitude of two obvious peaks around 35° and 260° are decreased comparing to the result shown in Figure 6a. It means that the multiple sound sources localization process will becomes more robust after post-processing. More details about the post-processing are described in Section 3.3.

### 3.3. Post-Processing

**Figure 7:**
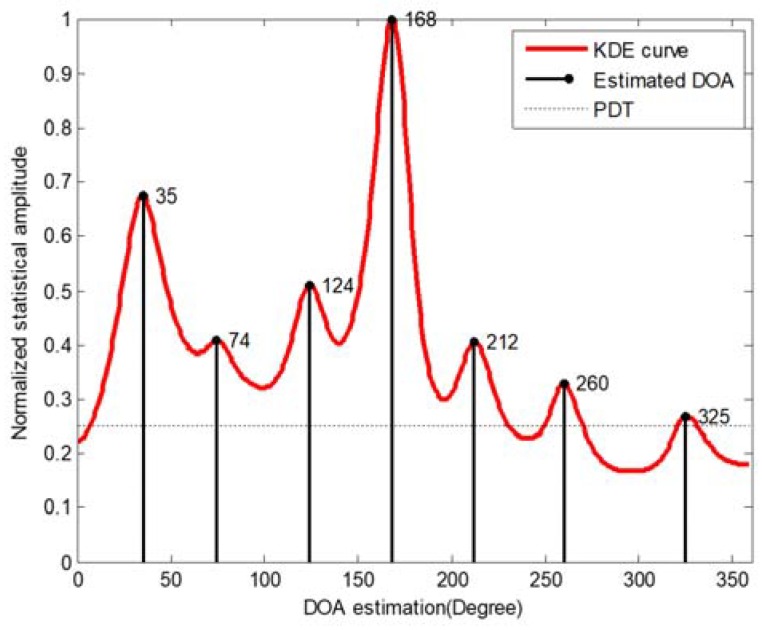
The result of peak searching over KDE curve.

The post-processing consists of two steps which are kernel density estimation (KDE) and peak searching. For obtaining a smooth estimated envelope curve of DOA histogram, KDE is selected in this paper. KDE belongs to a non-parametric estimation method for probability density function estimation. The sooth estimated curve can be generated using a series of observation points (i.e., DOA estimates). It means that the probability density function of a series of observation points can be estimated by KDE. If we suppose $\left\{y_{1},\;y_{2},\ldots ,y_{L_{K}}\right\}$ represents $L_{K}$ independent and identically distributed sample which belongs to a certain distribution with a density function f(·), the estimated shape of this density function $\hat{f}\left(\cdot \right)$ can be obtained from its kernel density estimator, which is given by:

(8) $$\;\hat{f}\left(y\right)=\frac{1}{L_{K}\cdot h}\overset{L_{K}}{\underset{i=1}{\sum }}K\left(\frac{y-y_{i}}{h}\right)\;$$

where K(·) is kernel function and h(h>0) is a smoothing parameter usually referred as the bandwidth. The input is all selected DOA estimates (i.e.,$\;{\hat{\mu }}^{\left(I\right)}\left(n,k\right)$) and the output is estimated a KDE curve by (8). The KDE curve of the DOA histogram shown in Figure 6b is represented by the red curve in Figure 7. It can be observed that each peak of DOA histogram is corresponding to a local maximum value in the KDE curve.

After obtaining the KDE curve, peak searching processing is exploited for the KDE curve to determine the estimated azimuth of sound sources. The core idea of peak searching is to search the local maximum value in the KDE curve of a DOA histogram. There are two important thresholds need to be set in the peak searching process, which are the peak detection threshold $\varepsilon_{p}$ (PDT) and the minimum difference threshold $\varepsilon_{a}$. The $\varepsilon_{a}$ is used to determine whether two adjacent local maximum values belong to the same sound source azimuth and the $\varepsilon_{p}$ is used to determine whether a local maximum value can be considered as a peak. Meanwhile, it should be noted that each peak is regarded as a sound source. Therefore, the peak searching process completes the task of sound sources counting at the same time. The result of the peak searching is shown in Figure 7 where the data is the same as Figure 1. It can be clearly seen that there are seven accurate peaks around the DOAs of real sources, i.e., all the estimated DOAs are almost equal with the real ones. Moreover, compared with Figure 1, not only can the sound source azimuths which are lost in Figure 1 be found in the DOA histogram obtained through the proposed SDSCR algorithm, but also the localization accuracy of other sound source azimuths is improved. 

**Figure 8:**
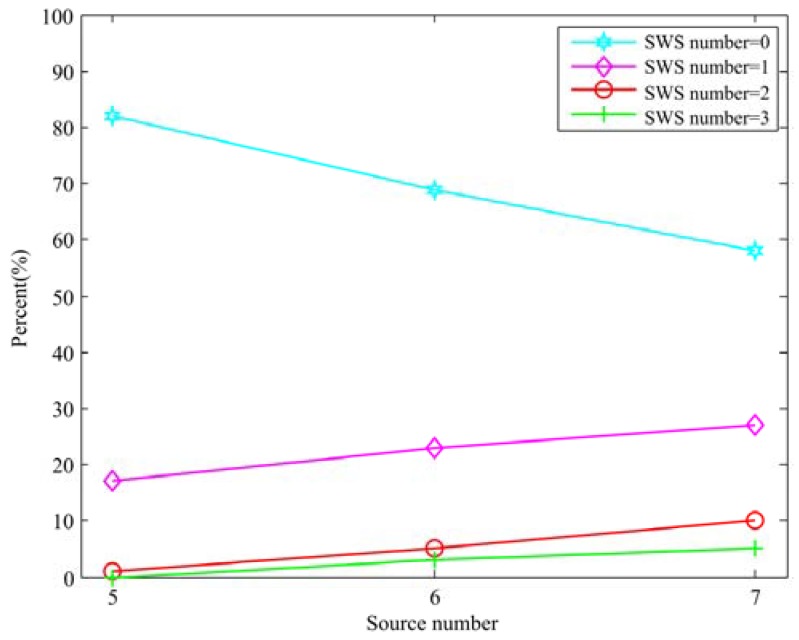
Statistical analysis for the existence of SWS in 30° angle between sources scenario exploit SDSCR algorithm.

Additionally, a statistical analysis for the SWS was taken for verifying the validity of proposed method. We exploited the same data which were used for the SWS counting in Figure 2b (angle between sources was set as 30°). The results of the SWSs number recount are shown in Figure 8. It can be observed that the number of SWSs with different sound sources number has decreased significantly compared with Figure 2b. When the sources number is five, the existence probability of one SWS drops from 38% to 17% which is more than half. It should be noted that the probability of no SWS has achieved different degrees of increasing in different sources number. More specifically, when the sources number was seven, the probability of no SWS rises from nearly 30% to nearly 60%. From the above results, the proposed localization method effectively makes the number of SWSs decreasing to reduce the impact of the presence of the SWS on localization results, and improves the localization accuracy compared with the conventional SSZ-based method. A more detailed evaluation of the proposed method is presented in next section. 

## 4. Results and Discussion

In this section, the experiments were conducted to evaluate the performance of the proposed approach in simulated and real environments. We compared the performance of the proposed method with the SSZ-based method [28] from two aspects which were accuracy of DOA estimation and sources counting respectively. The CMA-based method [27] was employed as a reference method in the evaluation of sources counting due to the model having achieved an approximately perfect performance in the sources counting.

For the DOA estimation case, both sources number and angle between sources were considered to value the accuracy of the proposed method and the SSZ-based method. We conducted the evaluation on the accuracy by mean absolute estimated error (MAEE) with different sources number and angle between sources in the anechoic room and reverberation room were tested, respectively. For the sources counting case, the percent of correct estimated number both in the anechoic and reverberation room were calculated for evaluating the performance of the proposed method, SSZ-based method and CMA-based method. For different test conditions, the experimental parameters and their corresponding values are listed in Table 2.

### 4.1. The Evaluation of Localization Performance in Simulated Environments

For the evaluation of localization performance, both the DOA estimation and sources counting need to be evaluated. The evaluation of DOA estimation was conducted in 4 different scenarios, i.e., Anechoic room, Room 1, Room 2, and Room 3. The parameters of these rooms are listed in Table 3. NTT is a speech database containing various speakers of different countries. The Chinese speech sub-data-base in NTT was employed as the test database in this section. We simulated the soundfield microphone system with the ROOMSIM package [29]. Both in the anechoic and reverberation room, the length of the simulated room was 6.25 m, the width was 3.75 m, and the height was 2.5 m. For the anechoic room, the sound sources number was 5 to 7 and the angle between sources were spaced by 10° from 30° to 50°. For the reverberation room, the sound sources number and the angle between sources were 3 to 5 and 50°, respectively. All sound sources were distributed on a circle with 2 m diameter, which were at the same height with the soundfield microphone and elevation angles were equal to 0°. We selected 72 and 36 different orientations for each sound sources number and angle between sources to evaluating localization accuracy of proposed method in the anechoic and reverberation room respectively. Moreover, for testing the performance all around the microphone, it should be mentioned that the initial orientation was 0° of the first source and then shifted by 5° and 10° in the anechoic and reverberation room respectively.

The MAEE is utilized to measure the performance of the proposed method and SSZ-based method, which calculates the difference between the true DOA and the estimated DOA. The MAEE can be calculated by:

(9) $$\;\mathrm{MAEE}=\frac{1}{N}\overset{N}{\underset{i=1}{\sum }}\frac{1}{P_{max}}\overset{P_{max}}{\underset{j=1}{\sum }}\left|\mu_{ij}-{\hat{\mu }}_{ij}\right|\;$$

where $\mu_{ij}$ is the true DOA of the *j*th active source in the *i*th experiment, and ${\hat{\mu }}_{ij}$ is the corresponding estimated DOA. $P_{i}$ is the number of active sources, while ${\hat{P}}_{i}$ is the estimated number of sources in the *i*th experiment and *N* is the total number of experiments. $P_{max}=\max\left\{P_{i},{\hat{P}}_{i}\right\}$. The following cases should be noted in (10):

(10) $$\begin{array}{l} \left(a\right)\;\;\;\;\mathrm{if}\;P_{i}>{\hat{P}}_{i}:\;{\hat{\mu }}_{ik}=0{}^{\circ},\;k\in \left[{\hat{P}}_{i}+1,P_{i}\right]\\ \left(b\right)\;\;\;\;\mathrm{if}\;P_{i}<{\hat{P}}_{i}:\;\mu_{ik}=0{}^{\circ},\;k\in \left[P_{i}+1,{\hat{P}}_{i}\right]\; \end{array}$$

The MAEE is collected in a series of experiments proceeded in different scenarios.

#### 4.1.1. DOA Estimation in Anechoic Room

**Figure 9:**
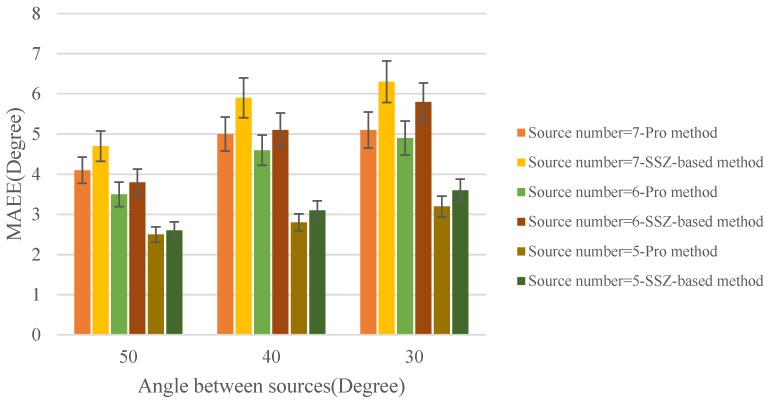
MAEE versus angle between sources in anechoic condition.

We measured the MAEE of the estimated DOA with different sources number and angle between sources in anechoic room for the proposed method and SSZ-based method [28] respectively. The results are shown in Figure 9 where can be concluded that both of two methods exhibits a good performance for multiple sound sources localization. Meanwhile, compared with SSZ-based method, the proposed method has lower MAEE in the same condition. 

From Figure 9, it can be found that proposed method provides a more accurate DOA estimation performance compared with the SSZ-based method. More specifically, when the angle between sources was set as 50° and the sound sources number were 7, the MAEE of the SSZ-based method and proposed method are 4.7° and 4.1°, respectively. Compared with the MAEE of the SSZ-based method, the MAEE of the proposed method decreasing 0.6° means that the localization accuracy improves by more than 10%. When the sound sources number is 7 and the angle between sources is 30°, compared with the SSZ-based method, the localization accuracy of proposed method improving by nearly 20%. Moreover, two obvious trends can be observed that the difference of the MAEE between two methods become larger with the increasing of the sound sources number or decreasing of the angle between sources, which means that the proposed method has a more significant improvement compared with the SSZ-based method in the performance of localization. In addition, this phenomenon is caused by a large amount of SWSs which is consistent with the obtained conclusion (i.e., with the sound sources number increasing or the angle between sources decreasing, there are more and more the number of SWSs existing among the soundfield microphone signals.) in Section 2. In conclusion, from the results shown in Figure 9, the proposed method achieves a better localization performance compared with the SSZ-based method.

#### 4.1.2. DOA Estimation in Reverberant Room

**Figure 10:**
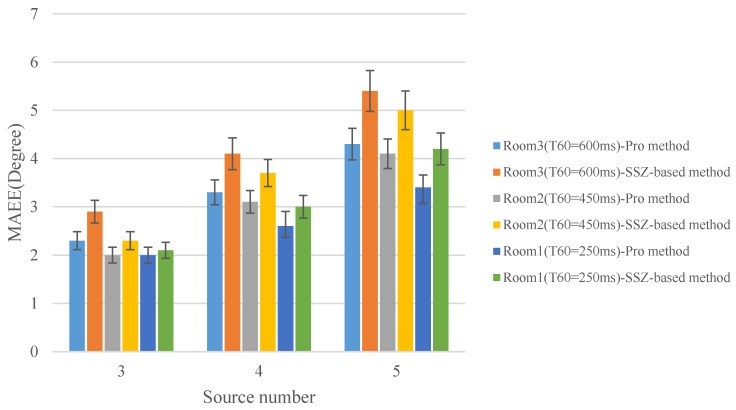
MAEE versus sound sources number in reverberant condition.

To investigate the robustness of the proposed method in reverberant environments, a set of experiments were proceeded in this section. The evaluation was conducted in four simulated rooms {Quiet room, Room 1, Room 2, Room 3}, and the parameters of them are listed in Table 3. The MAEE with different sources number in four rooms is shown in Figure 10.

As the reverberation time increases, although the DOA estimation accuracy of the proposed method and SSZ-based method decrease, however, the two methods still exhibit a reliable localization accuracy. Additionally, it can be observed that compared with the SSZ-based method, the proposed method has a lower MAEE in all experimental conditions which means that the proposed method achieves a better localization performance in the reverberant condition. With the sound sources number increasing, the same conclusions as in the case of without reverberation experiments can be drawn. Moreover, with the reverberation time increasing, compared with the localization accuracy of the SSZ-based method, the improvement of the proposed method for the localization accuracy is more obvious. In conclusion, the proposed method exhibits the ability to provide robustness under reverberation conditions.

#### 4.1.3. The Evaluation of Sources Counting

**Figure 11:**
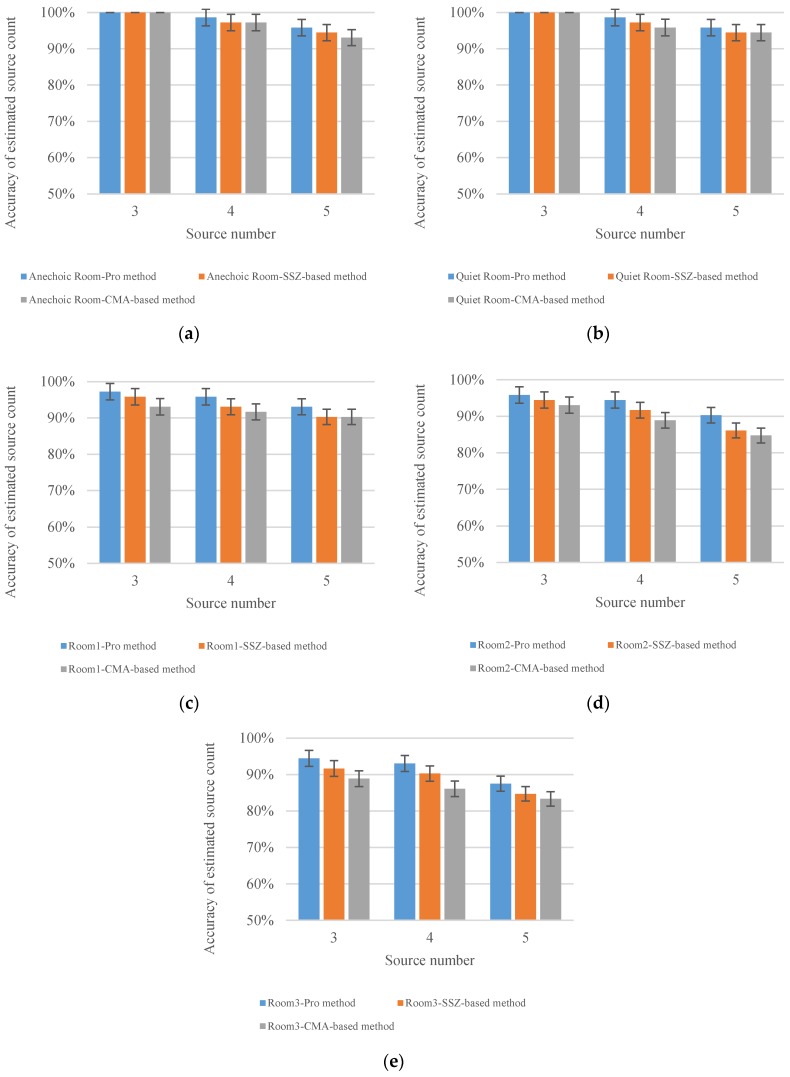
Objective comparison on accuracy among different sources counting algorithms under anechoic, quiet room and reverberant conditions with 95 % confidence intervals (**a**) Anechoic room; (**b**) Quiet room; (**c**) Room 1 (T60 = 250 ms); (**d**) Room 2 (T60 = 450 ms); (**e**) Room 3 (T60 = 600 ms).

Aiming to evaluate the accuracy of sources counting, the evaluation was conducted in five different simulated scenarios (i.e., Anechoic room, Quiet room, Room 1, Room 2 and Room 3). In each simulated room, we tested a certain number of simultaneously active sources ranging from 3 to 5. The angle between sources was set as 50°. The results of the estimated sources counting accuracy with 95% confidence intervals are shown in Figure 11. 

It could be seen from Figure 11a,b that when sources number equal 3, the proposed method and two comparison methods exhibit 100% estimated sources counting accuracy under both anechoic room and quiet room. When the sources number is equal to 4 and 5, three methods still show a good performance in the estimated sources counting accuracy, but the accuracy of the proposed method is slightly higher than the SSZ-based and CMA-based method. Figure 11c–e show that as the reverberation time increases, the estimated sources counting accuracy of the three methods decreases, but the proposed method still shows better sources counting accuracy than the SSZ-based and CMA-based method. More specifically, when the sources number equal to 5 and reverberation time is 450 ms, the sources counting accuracy of SSZ-based method and CMA-based method are both under 90%, while the sources counting accuracy of proposed method can still reach to 90%. A conclusion can be drawn that the proposed method is superior to SSZ-based and CMA-based method in the accuracy of sources counting.

### 4.2. The Evaluation of Localization Performance in Real Environments

After showing the evaluation results in simulated environments, the proposed SCSDR algorithm is further evaluated using soundfield microphone signals recorded in a real meeting room with an reverberation time approximately equal to 300 ms. The room is of dimension 6.5 m *×* 4.7 m *×* 2.8 m. The soundfield microphone (Twirling 720 VR Audio Recorder) [35] was placed at the center of room at a height of 1.5m and all speakers (i.e., sound source) are 1.3 m from the soundfield microphone. Additionally, the signal to noise ratio in the room was estimated at 20 dB. We evaluated the performance of the proposed method for different simultaneously active speakers number and angle between speakers. More specifically, the number of speakers was set as two and three, and the angle between speakers was 60° and 120°. For each pair of experimental parameters (i.e., the speakers number and angle between speakers), we selected 12 different locations for evaluation. 

**Figure 12:**
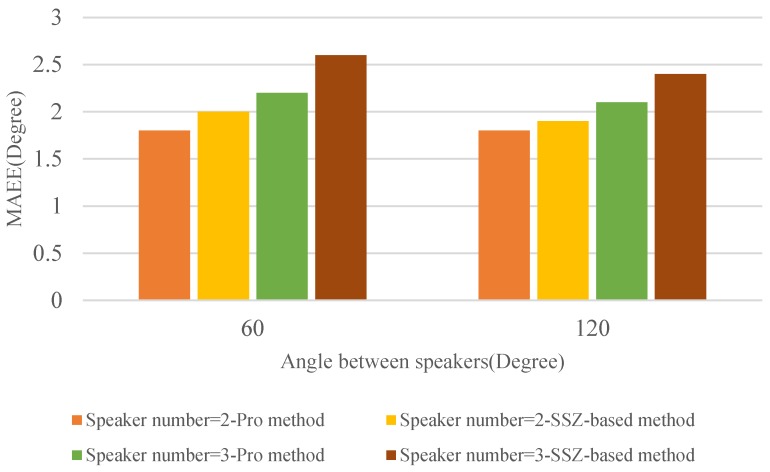
MAEE versus angle between speakers in real environments.

Results of evaluation for DOA estimation in the real environment are shown in Figure 12. From Figure 12, it can be found that both the proposed method and SSZ-based method provide a reliable result of localization in the real environment. Meanwhile, the performance of the proposed method is slightly better than that of the SSZ-based method. Compared with the results in simulated environments, we can find that the DOA estimation accuracy of the proposed method in the real environment is lower than that in the simulated environment. This phenomenon is caused by disturbing certain parameters such as ambient noise in the meeting room and reflection of objects (such as the desktop etc.).

## 5. Conclusions

In this paper, we found that SWSs, which are difficult to be detected by the conventional SSZ-based method, are ubiquitous when multiple sound sources occur simultaneously. The missed detection of SWSs degrades the performance of the SSZ-based method both in DOA estimation and sources counting. In order to find out the SWSs and improve the localization accuracy, we proposed a SDSCR algorithm for multiple sound sources localization. The SDSCR algorithm exploits joint intra-frame and inter-frame SDS discrimination to classify DOA estimates of all T-F bins into two categories (i.e., SWS components and SDS components). After the operation that retains SWS components and removes SDS components, the SWSs can be detected in histogram Statistics. The results show that the proposed method has a higher accuracy of DOA estimation as well as sources counting compared with the conventional SSZ-based method, and that it is robust in a reverberant environment. In future work, we will investigate the performance of the proposed method in various scenarios involving more sources with closer DOAs. 

## Figures and Tables

**Table 1:** Summary of the sound source localization method.

Method	Reference	Comment
TDOA	[13,14,15]	Employing excessive microphones to improve the reliability
MUSIC, ESPRIT	[17,18,19]	Microphones number more than sources number
SRP	[20]	High computational complexity
ICA	[16,21,22,23]	Employing directional sparsity of sound sources
SCA	[24,25,26]	Employing W-DO property
CMA-based	[27]	Need excessive microphones for multi-sources localization
SSZ-based	[28]	Instability for multi-sources localization

**Table 2:** Experimental parameters.

Parameter	Notation	Value
Sampling frequency of speech source	*fs*	16 kHz
Source distance	*r*	1 m
STFT length	*K*	2048
T-F zone width		64
Overlapping in frequency		50%
SSZ detection threshold	ε	0.1
Minimum difference threshold	$\;\varepsilon_{a}$	20°
Minimum distance threshold	$\;\varepsilon_{b}$	1
Minimum quantity threshold	$\;\varepsilon_{c}$	0.1
Peak detection threshold	$\;\varepsilon_{p}$	0.2
Length of data		1s

**Table 3:** Parameters of testing rooms.

Simulated Room	Reverberation Time
Anechoic Room	0 ms
Quiet Room	40 ms
Room 1	250 ms
Room 2	450 ms
Room 3	600 ms
